# Are we in the GI tract? Trachealization of the esophagus in a 10‐year‐old boy without eosinophilic esophagitis

**DOI:** 10.1002/jpr3.12019

**Published:** 2023-12-27

**Authors:** Geoffrey Daves, Aakash Goyal, Shannon Kelley

**Affiliations:** ^1^ Pediatric Gastroenterology University of Texas Southwestern Dallas Texas USA

**Keywords:** coagulative necrosis, esophagus, eosinophilic esophagitis

A 10‐year‐old male with a past medical history of allergic rhinitis and multiple food allergies was referred to our clinic for generalized abdominal pain and hematochezia for 6 weeks. Upon review of systems, the patient denied dysphagia, odynophagia, coughing, choking, toxic ingestion, drinking hot liquids or vomiting. To further evaluate the prolonged history of abdominal pain and potential source of bleeding, an upper and lower endoscopy was performed. Upper endoscopy revealed prominent mucosal rings extending nearly the entire length of the esophagus. Colonoscopy biopsies were normal. Endoscopic features of eosinophilic esophagitis and lymphocytic esophagitis, which tend to overlap,^1^ include linear furrows, mucosal rings, a small‐caliber esophagus, white plaques or exudates, and strictures.^2^ However, a definitive diagnosis is made based on histologic findings. Biopsies at all three levels of the esophagus were collected and to our surprise no eosinophils per high‐power field were revealed despite the trachealization of the esophagus. Specimen showed coagulative necrosis of the superficial esophageal epithelium without associated inflammation, a finding suggestive of injury, including thermal and caustic. There were also no histologic findings of lymphocytic infiltration to suggest lymphocytic esophagitis. Repeat endoscopy 4 months later on proton pump inhibitor acid suppression demonstrated gross normalization of the esophagus and normal findings on histology slides (Figures 1 and 2).

**Figure 1:**
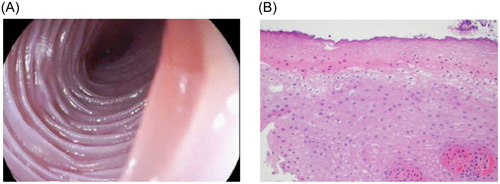
(A) Prominence of esophageal mucosal rings seen on esophagogastroduodenoscopy. (B) Esophageal biopsy with coagulative necrosis of the superficial layers.

**Figure 2:**
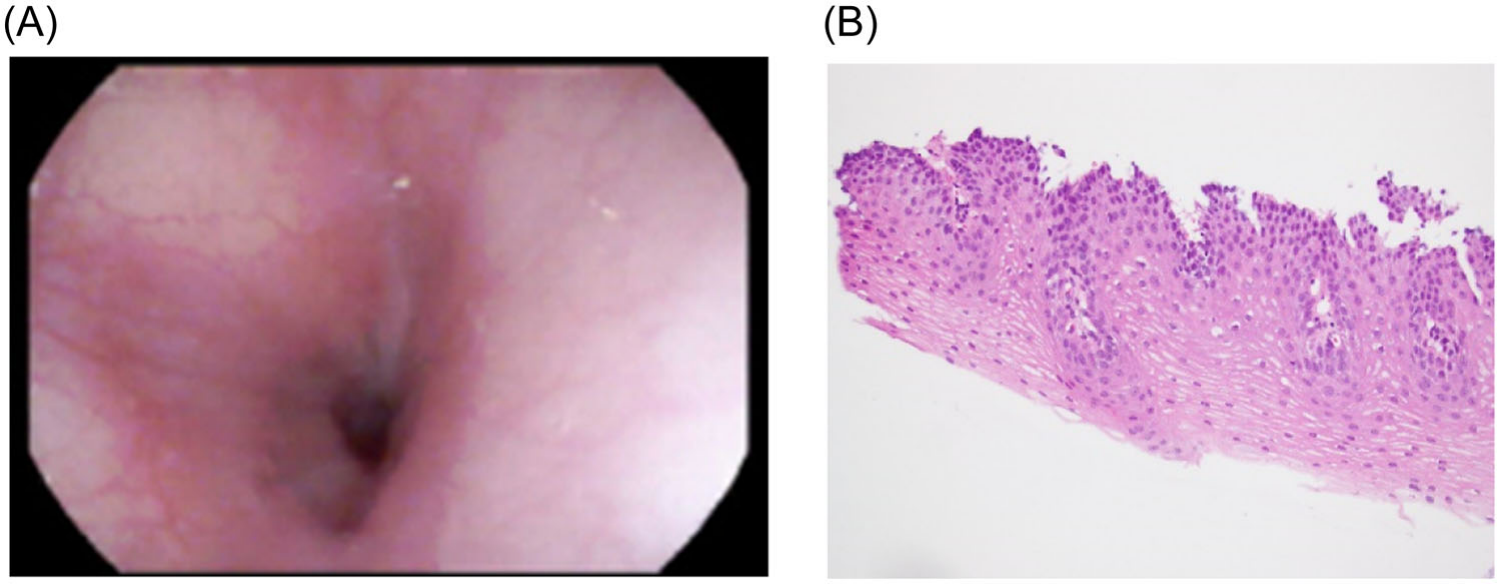
(A) Normal esophageal mucosa on repeat esophagogastroduodenoscopy 4 months later on proton pump inhibitor. (B) Normal esophageal biopsy.

## CONFLICT OF INTEREST STATEMENT

The authors declare no conflicts of interest.

## ETHICS STATEMENT

Informed consent was obtained from the patient's family.
